# Preclinical study of cinobufagin as a promising anti-colorectal cancer agent

**DOI:** 10.18632/oncotarget.13519

**Published:** 2016-11-23

**Authors:** Xing-sheng Lu, Yin-biao Qiao, Ya Li, Bo Yang, Min-bin Chen, Chun-gen Xing

**Affiliations:** ^1^ Department of General Surgery, The Second Affiliated Hospital of Soochow University, Suzhou, China; ^2^ Department of General Surgery, Suzhou Municipal Hospital, Suzhou, China; ^3^ Department of Hepatobiliary Surgery, The Third Hospital Affiliated to Soochow University, Changzhou City, Jiangsu, China; ^4^ Institute of Neuroscience, Soochow University, Suzhou, China; ^5^ Department of Medical Oncology, Kunshan First People's Hospital Affiliated to Jiangsu University, Kunshan, China

**Keywords:** colorectal cancer (CRC), cinobufagin (CBG), ER stress, mTOR and apoptosis

## Abstract

Here, we assessed the anti-colorectal cancer (CRC) cell activity of cinobufagin (CBG). We found that CBG exerted potent cytotoxic and anti-proliferative activity against CRC lines (HCT-116 and HT-29) and primary human CRC cells. Meanwhile, it activated apoptosis, and disrupted cell-cycle progression in the cells. At the signaling level, CBG treatment in CRC cells provoked endoplasmic reticulum stress (ER stress), the latter was evidenced by caspase-12 activation, CHOP expression, as well as PERK and IRE1 phosphorylations. Contrarily, the ER stress inhibitor salubrinal, the caspase-12 inhibitor and CHOP shRNA remarkably attenuated CBG-induced CRC cell death and apoptosis. Further, CBG in-activated mammalian target or rapamycin complex 1 (mTORC1), which appeared responsible for proliferation inhibition in CRC cells. Introduction of a constitutively-active S6K1 (“ca-S6K1”) restored proliferation of CBG-treated CRC cells. Finally, CBG intraperitoneal injection suppressed HCT-116 xenograft tumor growth in the nude mice. CHOP upregulation and mTORC1 in-activation were also noticed in CBG-treated HCT-116 tumors. The results of this preclinical study suggest that CBG could be tested as promising anti-CRC agent.

## INTRODUCTION

Colorectal cancer (CRC) is a major threat to human health, and it ranks one of leading causes of cancer-related mortalities in the world [1–3]. Conventional chemotherapies aren't able to kill CRC cells with pre-existing and/or acquired resistances [4]. Therefore, our group [5–10] and others are focusing on exploring novel and more potent anti-CRC agents [11]. Treatment cancer cells with Traditional Chinese Medicine (TCM) has been a research focus for many years [12]. One of these TCM, Chansu, is extracted from parotoid glands of the Chinese toad [13]. In ancient China and other Asian countries, Chansu has been widely utilized for the treatment of inflammation, anaesthesia and arrhythmia [13]. Cinobufagin (CBG) is a primary and active component from Chansu [14]. Recent preclinical studies have tested its anti-cancer activity [15–17]. However, its potential effect in CRC cells has not been extensively studied. More importantly, the underlying signaling mechanisms of CBG-mediated cancer cell killing effect are largely unknown.

Endoplasmic reticulum (ER) is the organelle that is key the synthesis, post-translational modification, proper folding, and maturation of new proteins [18, 19]. Interruption normal ER functions, *i.e.* by anti-cancer drugs, could result in pathological ER stress [18, 19], causing several unfolded protein responses (UPR) [20]. ER stress could lead to up-regulation of ER chaperones, including pro-apoptotic C/EBP homologous protein (CHOP) [21] and many others. Several ER membrane receptors, including double-stranded RNA-activated protein kinase (PKR)-like ER kinase (PERK), activating transcription factor 6 (ATF6) and inositol-requiring enzyme 1 (IRE1), could act as the sensors of ER stress [22]. In the current study, we provided evidences to show that CBG-induced CRC cell death is associated with ER stress activation.

## RESULTS

### Cinobufagin (CBG) exerts potent cytotoxic and anti-proliferative activity against human CRC cells

**Figure 1:**
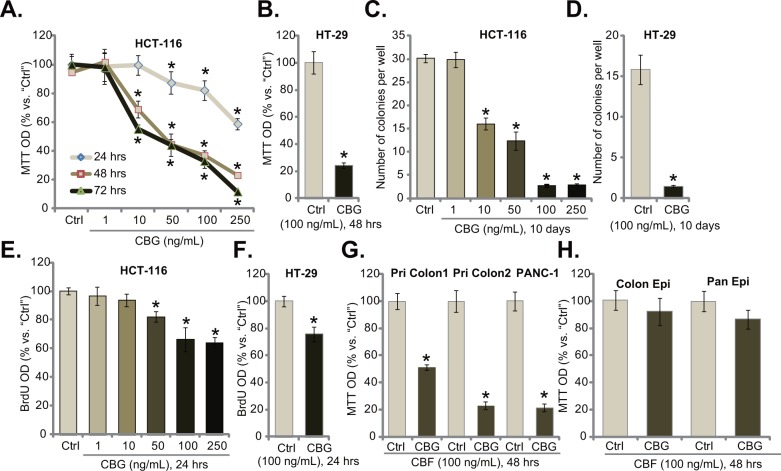
Cinobufagin (CBG) exerts potent cytotoxic and anti-proliferative activity against human CRC cells Listed cancer cells or non-cancerous epithelial cells were treated with/out designated concentrations of cinobufagin (CBG, 1-250 ng/mL), cells were further cultured for indicated time; Cell survival was tested by MTT assay **A**, **B**, **G** and **H.** and clonogenicity assay **C** and **D.**; Cell proliferation was tested by the BrdU ELISA assay **E** and **F.** Experiments in this figure were repeated five times, with similar results obtained. n=5 for each repeat. “Ctrl” stands for untreated control group (Same for all figures). * *****p***** < 0.05 vs. group of “Ctrl”.

To study the potential effect of CBG on CRC cells, HCT-116 CRC cells [6] were cultured in complete medium, and were treated with designated concentrations (1-250 ng/mL) of CBG. MTT cell viability assay results demonstrated that CBG dose-dependently inhibited HCT-116 cell survival (Figure 1A). CBG's IC50, the concentration that inhibited 50% of HCT-116 cell survival, was less than 50 ng/mL at 48 and 72 hours (Figure 1A). The lowest concentration of CBG (1 ng/mL) failed to inhibit HCT-116 cell survival (Figure 1A). Further, CBG also displayed a time-dependent response in inhibiting HCT-116 cells (Figure 1A). As early as 24 hours after CBG (50-250 ng/mL) treatment, a significant viability reduction was noticed, and it was more dramatic at 48 and 72 hours (Figure 1A). Notably, CBG (100 ng/mL, 48 hours) was also cytotoxic to HT-29 CRC cells (Figure 1B). Clonogenicity assay results demonstrated that CBG effectively decreased the number of viable colonies of HCT-116 cells (Figure 1C) and HT-29 cells (Figure 1D), further confirming its cytotoxicity against CRC cells. As shown in Figure 1E and 1F, CBG was also anti-proliferative when added to HCT-116 cells (Figure 1E, a dose-dependent response was observed) and HT-29 cells (Figure 1F). The BrdU OD was decreased in CBG-treated CRC cells (Figure 1E and 1F). Notably, BrdU OD was normalized to the cell viability (MTT OD) to exclude the influence of cell death (Figure 1E and F, same for all the BrdU assays of the study).

The effect of CBG on other cancer cells was also analyzed. As shown in Figure 1G, in two primary human colon cancer cell lines (“Pri Colon-1/−2”), treatment with CBG (100 ng/mL, 48 hours) also significantly decreased cell survival. Meanwhile, same CBG treatment was also cytotoxic to PANC-1 pancreatic cancer cells (Figure 1G) [23]. Intriguingly, the CBG treatment (100 ng/mL, 48 hours) was somehow non-cytotoxic to the primary colon epithelial cancer cells (“Colon Epi”) and to the HPDE6c7 pancreatic epithelial cells (“Pan Epi”) (Figure 1H), these results indicated a selective cytotoxicity of CBG to cancerous cells.

### Cinobufagin (CBG) provokes apoptosis in CRC cells

**Figure 2:**
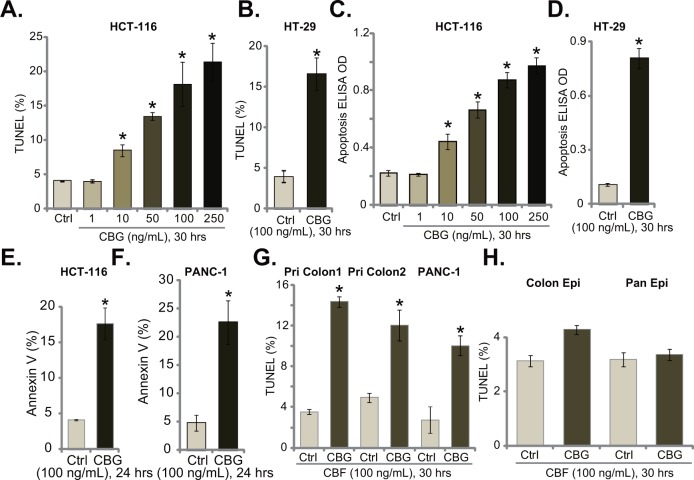
Cinobufagin (CBG) provokes apoptosis in CRC cells Listed cancer cells or non-cancerous epithelial cells were treated with/out designated concentrations of cinobufagin (CBG, 1-250 ng/mL), cells were further cultured for indicated time; Cell apoptosis was tested by TUNEL staining assay **A**, **B**, **G** and **H.**, histone-DNA ELISA assay **C** and **D.** and Annexin V FACS assay (**E** and **F.**, quantified results were presented at the right panels). Experiments in this figure were repeated three times, with similar results obtained. *p < 0.05 vs. group of “Ctrl”.

Next, we tested CBG's effect on CRC cell apoptosis, which was tested by previously described apoptosis assays [5–10]. TUNEL staining assay (Figure 2A and 2B), Histone-DNA ELISA assay (Figure 2C and 2D) and Annexin V FACS assay (Figure 2E and 2F) results demonstrated that CBG, at tested concentrations (10-250 ng/mL) efficiently provoked apoptosis in both HCT-116 cells and HT-29 cells (Figure 2A–2F). The TUNEL-positive cells (Figure 2A and 2B), the apoptosis ELISA OD (Figure 2C and 2D) and Annexin V percentage (Figure 2E and 2F) were all increased significantly following CBG (10-250 ng/mL) treatment in CRC cells. Further, TUNEL assay results showed that CBG (100 ng/mL) activated apoptosis in two lines of primary colon cancer cells (“Pri Colon-1/−2”) as well as in PANC-1 pancreatic cancer cells (Figure 2G). On the other hand, no profound apoptosis was observed in CBG (100 ng/mL)-treated colon epithelial cells (“Colon Epi”) and HPDE6c7 pancreatic epithelial cells (“Pan Epi”) (Figure 2H). These results clearly show that CBG provokes apoptosis in CRC cells.

### Cinobufagin (CBG) disturbs CRC cell cycle progression

**Figure 3:**
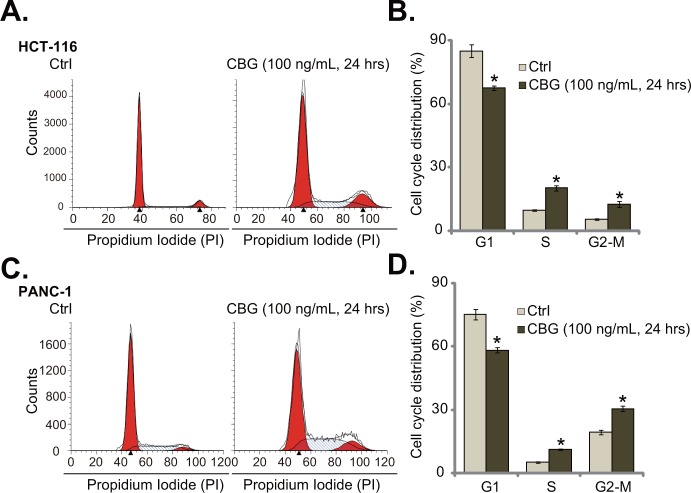
Cinobufagin (CBG) disturbs CRC cell cycle progression HCT-116 cells and PANC-1 cells were treated with/out cinobufagin (CBG, 100 ng/mL), cells were further cultured for 24 hours; Cell cycle distribution was shown **A** and **C.**; Data of three set repeats were quantified **B** and **D.** * *****p***** < 0.05 vs. group of “Ctrl”.

The effect of CBG on CRC cell cycle progression was also tested. As shown in Figure 3A, following treatment of CBG (100 ng/mL, 24 hours) in HCT-116 cells, the percentage of G1 phase cells was significantly decreased. Correspondingly, the percentages of S and G2-M phase cells were increased (Figure 3A). Quantified results in Figure 3B further demonstrated G2-M arrest in CBG-treated HCT-116 cells (Figure 3B). Same experiments were also performed in PANC-1 cells, and similar G2-M arrest was noticed (Figure 3C and 3D). These results demonstrate that CBG induces G2-M arrest in the cancer cells.

### CBG-mediated CRC cell death is associated with ER stress activation

**Figure 4:**
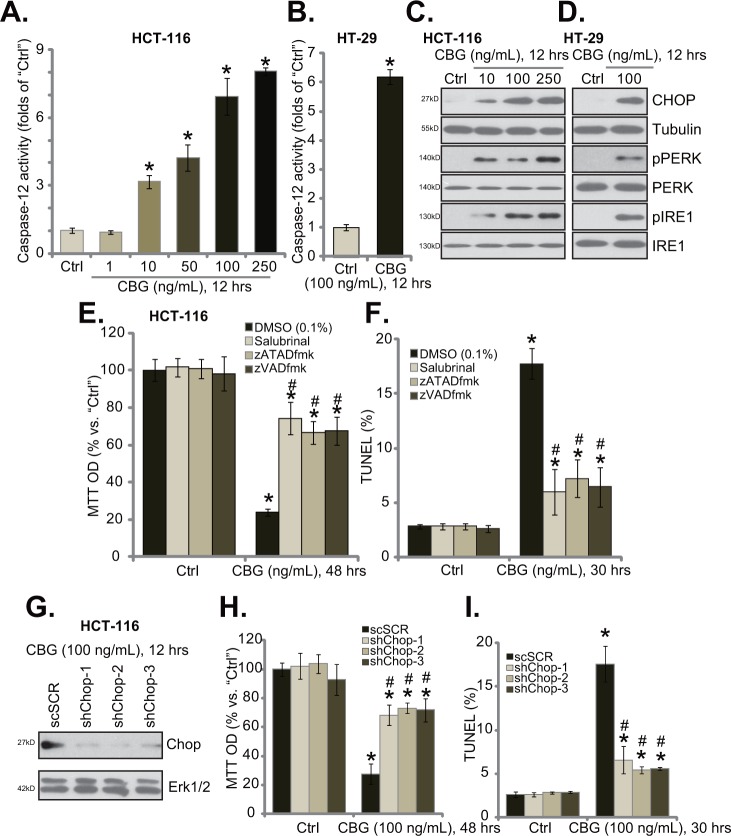
CBG-mediated CRC cell death is associated with ER stress activation Listed cancer cells were treated with/out designated concentrations of cinobufagin (CBG, 1-250 ng/mL), cells were further cultured for indicated time; Caspase-12 activity **A** and **B.** and expressions of listed ER stress proteins **C** and **D.** were tested. HCT-116 cells were pre-treated 1 hour with salubrinal (10 μM), zATADfmk (50 μM), or zVADfmk (50 μM), followed by cinobufagin (CBG, 100 ng/mL) stimulation, cells were further cultured for indicated time; Cell viability (**E.**, MTT assay) and apoptosis (**F.**, TUNEL staining assay) were tested. Stably HCT-116 with scramble-shRNA (“shSCR”) or CHOP-shRNA (“shCHOP-1/−2/−3”) were treated with cinobufagin (CBG, 100 ng/mL) for indicated time; CHOP expression **G.**, cell survival **H.** and apoptosis **I.** were tested. Experiments in this figure were repeated four times, with similar results obtained. * *****p***** < 0.05 vs. group of “Ctrl”. ^#^ *****p***** < 0.05 vs. CBG treatment group of “DMSO” (E and F) or “shSCR” (H and I).

When analyzing potential caspases that were possibly activated by CBG, we found that caspase-12 activity was significantly increased in CBG-treated HCT-116 cells and HT-29 cells (Figure 4A and 4B). Activation of caspase-12 is a characteristic marker of ER stress apoptosis pathway [24, 25], we thus tested other ER stress-associated proteins in CBG-treated cells. Western blot results demonstrated that treatment of HCT-116 cells (Figure 4C) and HT-29 cells (Figure 4D) with CBG (10-250 ng/ml) induced CHOP expression, PERK and IRE1 phosphorylations, indicating ER stress activation [18, 20]. Remarkably, the ER stress inhibitor salubrinal [26], the caspase-12 inhibitor zATADfmk as well as the pan caspase inhibitor zVADfmk largely inhibited CBG (100 ng/ml)-induced HCT-116 cell death (Figure 4E) and apoptosis (Figure 4F), indicating the requirement of ER stress in mediating CBG's cytotoxicity.

To further support our hypothesis, shRNA strategy was applied to knockdown CHOP, a key protein of ER stress apoptosis pathway [21]. The three CHOP shRNAs with non-overlapping sequences were applied, and each of them efficiently downregulated CHOP in CBG-treated HCT-116 cells (Figure 4G). Remarkably, CHOP shRNA knockdown largely attenuated CBG's killing of HCT-116 cells (Figure 4H and 4I). Thus, CHOP and ER stress activation mediated CBG-induced CRC cell death and apoptosis.

### CBG inhibits mTORC1 activation in CRC cells

**Figure 5:**
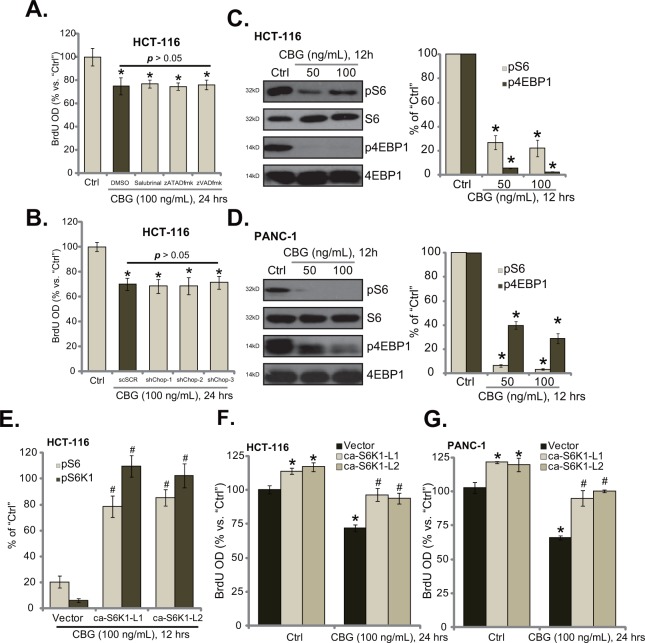
CBG inhibits mTORC1 activation in CRC cells HCT-116 cells were pre-treated 1 hour with salubrinal (10 μM), zATADfmk (50 μM), or zVADfmk(50 μM), followed by cinobufagin (CBG, 100 ng/mL) stimulation for applied time; Cell proliferation was tested by BrdU ELISA assay **A.** Stably HCT-116 with scramble-shRNA (“shSCR”) or CHOP-shRNA (“shCHOP-1/−2/−3”) were treated with cinobufagin (CBG, 100 ng/mL) for indicated time; Cell proliferation was tested **B.** HCT-116 cells or PANC-1 cells were treated with/out cinobufagin (CBG, 50/100 ng/mL) for 12 hours, expressions of listed proteins were shown (**C** and **D.**, data of four repeats were quantified at right panels). Stably HCT-116 cells **E** and **F.** or PANC-1 cells **G.**, expressing empty vector (“pGCL-flag-puro”) or the constitutively-active S6K1 (T389E, “ca-S6K1”, two different lines “L1/L2”), were treated with/out cinobufagin (CBG, 100 ng/mL) for indicated time; Relative S6 and S6K1 phosphorylations (% *vs.* “Ctrl” cells) were shown (E, integration of three repeats); Cell proliferation was tested by BrdU ELISA assay (F and G). Experiments in this figure were repeated four times, with similar results obtained. * *****p***** < 0.05 vs. group of “Ctrl”. ^#^*****p***** < 0.05 vs. “CBG” treatment group of “Vector” cells (E-G).

Interestingly, we showed that the ER stress inhibitor salubrinal and the caspase inhibitors (zATADfmk and zVADfmk) failed to reverse or attenuate cell proliferation inhibition by CBG in HCT-116 cells (Figure 5A). Similarly, CHOP shRNA knockdown in HCT-116 cells also didn't ameliorate CBG-induced proliferation inhibition (Figure 5B). These results indicated that ER stress apparently didn't participate in CBG-induced anti-proliferative action. Therefore, we tested several proliferation-associated signalings in CBG-treated cancer cells. Significantly, we found that CBG (50/100 ng/mL) treatment in HCT-116 cells largely inhibited mammalian target of rapamycin (mTOR) complex 1 (mTORC1) activation, the latter was evidenced by phosphorylations (“p”) of S6 and 4EBP1 [27, 28] (Figure 5C). Similar results were also obtained in PANC-1 cells, where CBG (50/100 ng/mL) largely inhibited S6 and 4EBP1 phosphorylations (Figure 5D).

To study the link between CBG-induced mTORC1 in-activation and proliferation inhibition, we introduced the constitutively-active S6K1 (T389E, “ca-S6K1”) [29] into HCT-116 cells, and two stably lines (“ca-S6K1-L1/2”) were established. Quantified results in Figure 5E demonstrated that ca-S6K1 restored S6 and S6K1 phosphorylations in CBG (100 ng/mL)-treated HCT-116 cells to almost control level. Consequently, cell proliferation was almost recovered by ca-S6K1 in HCT-116 cells (Figure 5F). Similarly in PANC-1 cells, introduction ca-S6K1 restored cell proliferation even with CBG (100 ng/mL) treatment (Figure 5G). Notably, ca-S6K1 alone also promoted proliferation of above cancer cells (Figure 5F and 5G), further confirming the positive role of mTOR in promoting cell proliferation. These results suggest that CBG-mediated proliferation inhibition in above cancer cells could be due to mTORC1 in-activation.

### Cinobufagin (CBG) inhibits HCT-116 tumor growth in nude mice

**Figure 6:**
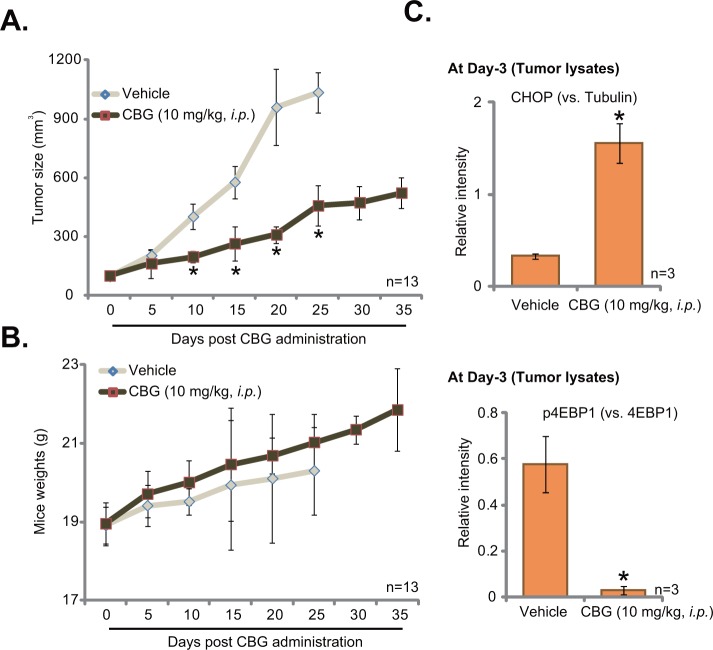
Cinobufagin (CBG) inhibits HCT-116 tumor growth in nude mice HCT-116 tumor-bearing nude mice (n=13 per group) were administrated with cinobufagin (CBG, 10 mg/kg, *i.p.*, daily) or vehicle control (“Vehicle”) for 20 consecutive days, tumor volumes (in mm^3^) **A.** and mice body weights (**B.**, in grams) were recorded every 5 days. At day three after initial CBG administration, three HCT-116 tumors per group were isolated, expressions of listed proteins in tumor lysates were tested by Western blot assay, and data were integrated and quantified **C.**; * *****p***** < 0.05 vs. group of “vehicle”.

To study the anti-cancer activity by CBG *in vivo*, the mouse xenograft tumor model was established. A significant number of HCT-116 cells were injected in the flanks of nude mice. Within three weeks, xenograft HCT-116 tumors were established. The growth curve results in Figure 6A demonstrated that CBG administration (10 mg/kg body weight, *i.p.*, daily) significantly inhibited HCT-116 tumor growth in the nude mice. The tumor volumes in CBG-treated mice were significantly lower than those of vehicle control mice (Figure 6A). Yet, the mice body weights in the CBG mice were not significantly different from that of vehicle control mice (Figure 6B), indicating the relative safety of the CBG regimen here. Notably, no apparent toxicities were noticed in the CBG-treated mice. Next, we wanted to know if the signaling changes by CBG *in vitro* were also achieved *in vivo*. At day three following initial CBG treatment, HCT-116 tumors were isolated, and expressions of above signaling proteins in tumor lysates were tested by Western blot assay. Quantified results in Figure 6C clearly demonstrated CHOP upregulation and p4EBP1 inhibition in HCT-116 tumors with CBG administration. Therefore, in line with the *in vitro* findings, CBG administration *in vivo* possibly also induced ER stress activation and mTORC1 in-activation in HCT-116 tumors.

## DISCUSSION

In the current study, we showed that CBG exerted cytotoxic, pro-apoptotic and anti-proliferative actions against established (HCT-116 and HT-29) and primary CRC cells. *In vivo*, CBG administration largely inhibited HCT-116 tumor growth in nude mice. ER stress activation and mTORC1 in-activation apparently mediated CBG's actions in CRC cells. Interestingly, ER stress inhibition, by adding salubrinal, zATADfmk, or CHOP shRNAs, didn't totally abolish CBG-mediated CRC cell death (Figure 4). Therefore, other mechanisms besides ER stress should also participate in CBG-induced killing of CRC cells. For example, it has been shown that CBG exerted cytotoxicity in multiple myeloma cells via activating ROS-mediated MAPK signaling pathway [30]. Qi *et al.,* showed that CBG activated Fas- and mitochondria-mediated pathways to kill hepatocellular carcinoma cells [16]. The study by Yu et al., suggested a pivotal role of p53 activation in mediating CBG-induced prostate cancer cell apoptosis [17]. Thus, it will be interesting to test the association between ER stress activation and these other cascades in CBG-treated CRC cells.

Although ER stress activation mediated CBG-induced CRC cell death, blockage of this pathway didn't bring back proliferation of CRC cells. Here, we suggested that mTORC1 inactivation could be important for CBG's anti-proliferative activity in CRC cells. Remarkably, introduction of the ca-S6K1 restored proliferation in CBG-treated CRC cells. Notably, there was no crosstalk between ER stress activation and mTORC1 in-activation in CBG-treated cells. Salubrinal, zATADfmk, or CHOP shRNAs had no effect on CBG-induced mTORC1 in-activation in CRC cells; Meanwhile, the ca-S6K1 also failed to inhibit ER stress activation by CBG. Therefore, these two signalings by CBG appeared parallel to each other, and exerted separated functions in CRC cells.

Intriguingly, we showed that CBG treatment failed to induce significant cytotoxicity to normal colon and pancreatic epithelial cells. Thus, CBG treatment was relatively safe to the normal cells. One possibility could be that mTORC1 (p4EBP1/pS6) was only hyper-activated in cancer cells, but was quite low in the epithelial cells. Another possibility is that CBG-induced ER stress activation somehow could only promote cancerous cell death, as ER function is often hyper-activated in cancer cells [20, 31]. Normal epithelial cells could then be tolerate to ER stress by CBG.

One important finding of this study is that CBG was also cytotoxic when added to PANC-1 pancreatic cancer cells, where ER stress activation and mTORC1 in-activation were similarly noticed. Therefore, it would be interesting to test CBG's anti-cancer activity against other solid tumor cells, and whether the mentioned two signalings (ER stress activation and mTORC1 in-activation) could also been observed in other cancer cells. Together, these preclinical results suggest that CBG could be a promising chemotherapeutic agent for CRC and possible other cancers.

## MATERIALS AND METHODS

### Chemicals and reagents

Cinobufagin (CBG) and the ER stress inhibitor salubrinal [26] were purchased from Sigma Chemicals (St. Louis, MO). The pan-caspase inhibitor (z-VAD-fmk) and the specific caspase-12 inhibitor (z-ATAD-fmk) [32] were purchased from Calbiochem (Darmstadt, Germany). All phosphorylation antibodies and their non-phosphorylation control antibodies were purchased from Cell Signaling Technology (Beverly, MA). All other antibodies were obtained from Santa Cruz Biotech (Santa Cruz, CA). The concentrations of agents applied and the treatment durations were chosen based on published literatures and results from our pre-experiments.

### Culture of established cell lines

As reported early [5–10], CRC lines HT-29 and HCT-116 were maintained in DMEM medium with 10% FBS, Penicillin/Streptomycin and 4 mM L-glutamine, in a CO_2_ incubator at 37°C. PANC-1 pancreatic cancer cells, purchased from the Cell Bank of Shanghai Institute of Biological Science (Shanghai, China), were cultured in above DMEM medium. HPDE6c7, an immortalized pancreatic epithelial cell line [33], was purchased from GuangZhou Jennio Biotech (Guangzhou, China). HPDE6c7 cells were cultivated in DMEM supplemented with 10% FBS. For all the cell lines, DNA fingerprinting and profiling were performed every 6 months to confirm the origin of the cell line, and to distinguish the cell line from cross-contamination. All cell lines were subjected to mycoplasma and microbial contamination examination. Population doubling time, colony forming efficiency, and morphology under phase contrast were also measured every 6 months under defined conditions to confirm the phonotype of cell line.

### Primary culture of colon cancer and epithelial cells

As previously described [8, 10], the fresh colon cancer tissues along with the surrounding normal epithelial tissues were separated carefully under the microscope. Tissues were thoroughly washed and minced, which were then mechanically dissociated and filtered via a 70 μm strainer. Single-cell suspensions were achieved by re-suspending cells in 0.15% (w/v) collagenase I (Sigma) containing medium. Cells were then cultured in the medium as described [10]. The study was approved by the institutional review board (IRB) of all authors' institutions. Procedures were conducted according to the principles expressed in the Declaration of Helsinki. Two male patients (55/61 year old) with primary colon cancer were enrolled. Written-informed consent was obtained from each participant.

### Methyl thiazol tetrazolium (MTT) assay

Cell viability was assessed by the MTT assay as described [7, 8, 34].

### Colony formation assay

As previously reported [8, 35], cells with applied treatment were suspended in agar-containing medium, which were then added on the top of a culture dish. After 10 days of incubation, the number of colonies were fixed, stained and manually counted.

### Annexin V FACS assay of cell apoptosis

After treatment, cells were washed and incubated with Annexin V-FITC (3 μg/mL, Invitrogen, Shanghai, China) and Binding Buffer (Invitrogen). A total of 10,000 cells of each sample were analyzed by flow cytometry in a FACS (Beckton Dickinson, Cytoflex, Shanghai, China). The percentage of Annexin V was utilized as a quantitative measurement of cell apoptosis.

### Histone-DNA Enzyme-linked immunosorbent assay (ELISA) assay

As previously described [8], cell apoptosis was quantified by Histone-DNA ELISA PLUS kit (Roche Applied Science, Shanghai, China).

### Western blot assay

Western blot assay was performed as previously described [5–10]. Blot intensity was quantified by ImageJ software (NIH).

### Caspase-12 activity assay

Following treatment of cells, cytosolic extracts (20 μg per treatment) were added to the caspase assay buffer [36] plus the caspase-12 substrate ATAD-7-AFC (Invitrogen). The amount of liberated AFC was tested by a spectrofluorometer (Thermo-Fisher, Shanghai, China).

### TUNEL staining assay

TUNEL (Terminal deoxynucleotidyl transferase dUTP nick end labeling) In Situ Cell Death Detection Kit (Roche, Shanghai, China) was applied to evaluate cell apoptosis. TUNEL percentage (TUNEL/DAPI×100%) was calculated from at least 100 cells per treatment.

### BrdU incorporation assay

Cells (0.25 × 10^5^ cells/well) were seeded in complete medium. The BrdU ELISA colorimetric assay kit (Cell Signaling) was applied to test cell proliferation [37].

### CHOP shRNA knockdown

Three lentiviral shRNAs targeting non-overlapping sequence of human *CHOP* sequence were designed, synthesized and validated by Genepharm Co. (Shanghai, China). Ten μL/mL of lentiviral shRNA was added to the HCT-116 cells for 48 hours; Stable HCT-116 clones expressing targeted-shRNA were selected by puromycin (5.0 μg/mL) for 4-6 passages. CHOP expression in the resistant colonies was detected by Western blot assay.

### The constitutively active S6K1 construct and transfection

The constitutively active S6K1 (T389E, “ca-S6K1-flag-puro”) and the empty vector (pGCL-flag-puro) were gifts from Dr. Chen's group [29]. The construct was transfected via Lipofectamine 2000 reagents (Invitrogen) [29]. Cells were subjected to puromycin (5.0 μg/mL) selection for 4-6 passages. “ca-S6K1” expression in the stable cells was again verified by Western blot assay.

### Xenograft assay

Five million HCT116 cells in PBS (200 μL) were subcutaneously injected into each mouse. Within three weeks, the xenografted tumors were established with the size around 100 mm^3^. The nude mice (aged 7-8 weeks, weighting 18-19 g) were equally divided into two groups: intraperitoneal (*i.p.*) injection and control. CBG was initially dissolved in ethanol and was then diluted in 10 % propylene glycol solution. The daily dose given to *i.p.* group was 10 mg/kg of body weight, while an equal amount of injection solution without CBG was given as the vehicle control. Mice were sacrificed when the tumor grew over 100 mm^3^. Tumor volumes were recorded, calculated via the following formula: π/6 × larger diameter × (smaller diameter)^2^. All studies were performed in accordance with the standards of ethical treatment approved by the Institutional Animal Care and Use Committee (IACUC).

### Statistical analysis

Data were presented as mean ± standard deviation (SD). Statistics were analyzed by one-way ANOVA using the SPSS 18.0 software (SPSS Inc., Chicago, IL). Significance was chosen as ***p*** < 0.05.
